# Number of positive lymph nodes affects outcomes in parotid adenoid cystic carcinoma

**DOI:** 10.3389/fonc.2023.1153186

**Published:** 2023-03-23

**Authors:** Feng Han

**Affiliations:** Department of Pediatric Dentistry, The First Affiliated Hospital of Zhengzhou University, Zhengzhou, Henan, China

**Keywords:** parotid gland, adenoid cystic carcinoma, number of positive lymph nodes, survival, AJCC

## Abstract

**Objectives:**

Survival significance of the number of positive lymph nodes (LNs) in parotid adenoid cystic carcinoma (ACC) remains unknown; thus, this study aimed to determine the impact of the number of positive LNs on the prognosis of parotid ACC.

**Methods:**

Patients with surgically treated parotid ACC were enrolled from the SEER database. The number of positive LNs was analyzed using three models (0 vs 1+, 0 vs 1 vs 2 vs 3 vs 4 vs 4 vs 5 vs 6+, 0/1 vs 2–4 vs 5+), its hazard ratios on disease specific survival (DSS) and overall survival (OS) were assessed using univariate and multivariate Cox analyses.

**Results:**

A total of 1,689 patients were included. In all models, the number of positive LNs was independently related to DSS and OS, model 3 had the highest C-index for DSS [0.83 (95% CI: 0.81–0.85)] and OS [0.82 (95% CI: 0.80–0.84)]. Compared with the 0/1 positive LN group, the 2–4 positive LN group had an HR of 2.81 (95% CI: 1.73–4.56) for DSS and 2.36 (95% CI: 1.58–3.54) for OS. The 5+ LN group had an HR of 20.15 (95% CI: 7.50–54.18) for DSS and 14.20 (95% CI: 5.45–36.97) for OS. No overlap existed in the 95% CI of the HR.

**Conclusions:**

The three prognostic categories based on the number of positive LNs (0/1 vs 2–4 vs 5+) could stratify the DSS and OS in parotid ACC without overlap.

## Introduction

Adenoid cystic carcinoma (ACC) is one of the most common malignancies among all parotid tumors. It is characterized by distant metastasis and perineural infiltration (1), making it remarkably different from other parotid cancers. Although neck nodal metastasis is relatively uncommon, it is still an important prognostic factor in parotid ACC (2). The neck nodal status for parotid cancer is currently deduced from head and neck squamous cell carcinoma (HNSCC), which is evidently inadequate. Meanwhile, the intraparotid lymph node (LN), which significantly affects prognosis, is not taken into consideration (3, 4). On the other hand, parotid cancer exhibits distinct differences in biology from HNSCC. Contralateral neck LN metastasis is extremely rare in parotid cancer, and this staging fails to distinguish the hazard ratio (HR) of four groups in relation to the prognosis (2, 5). In other words, the 95% confidence interval (CI) of a stage overlaps with the adjacent stages.

Novel LN stagings have been proposed based on the number of positive LNs and/or LN size (6, 7), in which the systems are determined according to the different cutoffs of quantitative LN burden. Both exhibit greater concordance than the current neck nodal classification. However, the two previous studies have analyzed data comprising all major salivary gland histologic types. Thus, whether the findings could be applied for parotid ACC is uncertain.

Therefore, this study aimed to clarify the impact of the number of positive LNs on the prognosis of parotid ACC.

## Patients and methods

### Study design

**Figure 1:**
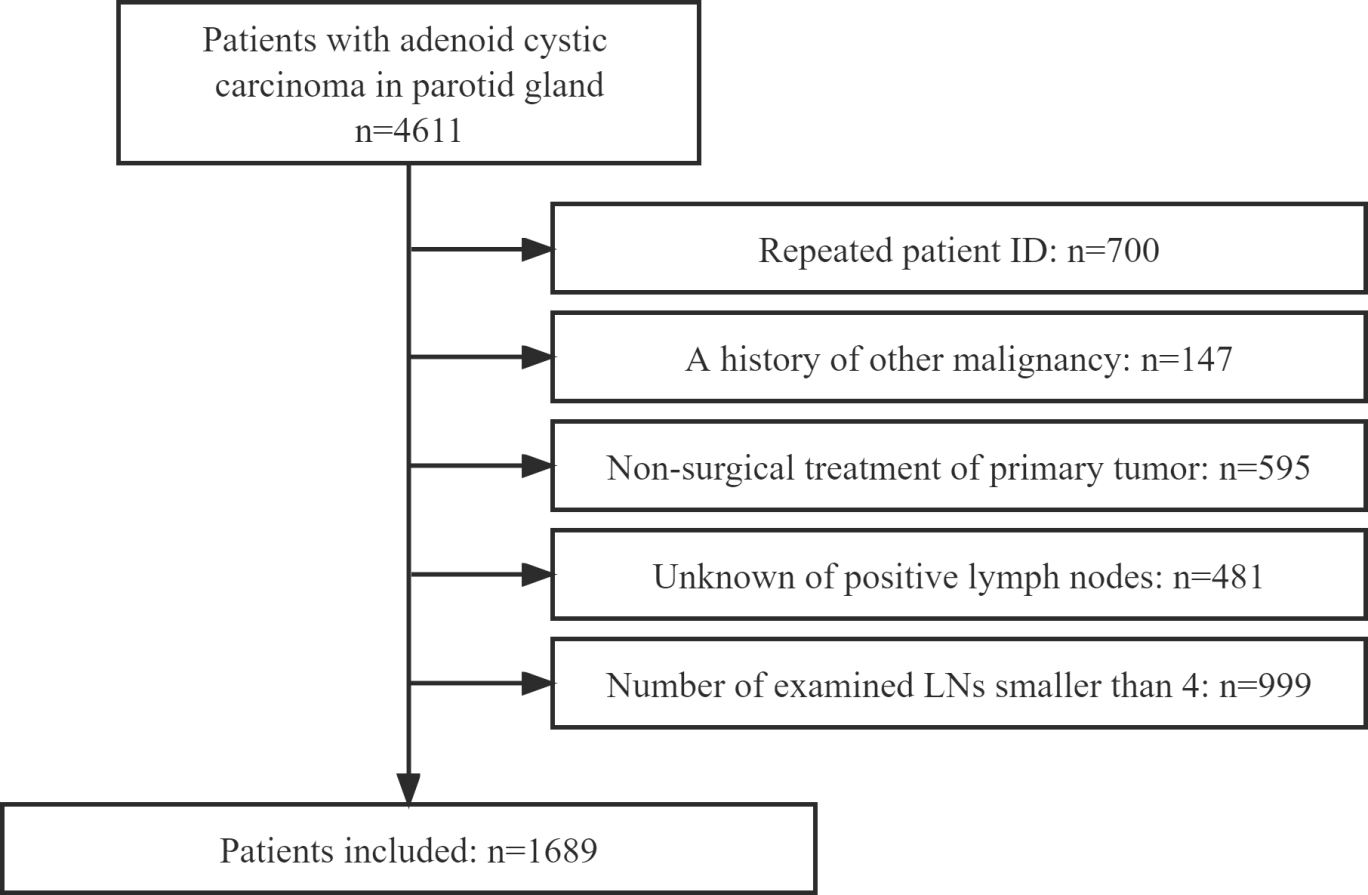
Flowchart of the enrolled patients.

All data was obtained from the Surveillance, Epidemiology, and End Results database, which provides information on cancer statistics to reduce the cancer burden among the United States population (8). The profiles of the patients diagnosed with parotid ACC between 2000 and 2019 were reviewed. Patients were excluded as follows: repeated patient ID; a history of other malignancy; non-surgical treatment of primary tumor; unknown number of positive LNs; and number of pathologically examined LNs is smaller than 4 (**Figure 1**). Information regarding age, sex, race, marital status, tumor size, tumor extension, grade, pathologic tumor stage based on the 8^th^ American Joint Committee on Cancer (AJCC) classification, extranodal extension (ENE), distant metastasis, operation type, radiation, chemotherapy, number of positive LNs, and follow-up were extracted and analyzed.

Ethical approval was not required because the data is publicly accessible.

### Variable definition

The disease grade was classified into low, moderate, and high. A low grade was defined as well differentiated; a moderate grade was defined as moderately differentiated; and a high grade was defined as poorly differentiated or undifferentiated. The tumor size was determined based on the Tumor Size Summary (2016+), Collaborative Stage tumor size (2004–2015), and extent of disease (EOD) 10-size (1988–2003). The tumor extension was defined as extracapsular invasion and evaluated based on the Derived EOD 2018 T (2018+), Collaborative Stage extension (2004–2015), and EOD 10-extent (1988–2003). The tumor stage was extrapolated based on the tumor size, tumor extension, and Derived AJCC classification. ENE was formulated based on Derived EOD 2018 N (2018+), Derived AJCC classification, RX Summ–Scope Reg LN Sur (2003+), EOD Regional Nodes (2018+), CS lymph nodes (2004–2015), and EOD 10 - nodes (1988–2003). Distant metastasis was confirmed using the Derived AJCC classification. The type of operation consisted of non-total and total parotidectomy and was decided based on the RX Summ–Surg Prim Site (1998+). The number of positive LN was calculated based on the Regional nodes examined (1988+), Regional nodes positive (1988+), and RX Summ–Scope Reg LN Sur (2003+). The time to surgery (TTS) was defined as the duration between the diagnosis and treatment.

### Statistical analysis

Missing data patterns were evaluated on whether they occur at random using the method previously introduced and imputed using multiple imputation by fully conditional specifications, which was implemented using multiple imputation by chained equations (7, 9).

The primary outcome variable was disease-specific survival (DSS) and overall survival (OS). The time of DSS was calculated from the date of surgery to the date of cancer-caused death or last follow-up. Meanwhile, the time of OS was calculated from the date of surgery to the date of overall death or last follow-up.

Three models were constructed using different cutoffs for the number of positive LNs to detect the optimal cutoff. In model 1, the impact was compared between the 0 and 1+ groups. In model 2, the impact was analyzed among the 0, 1, 2, 3, 4, 5, and 6+ groups. In model 3, the impact was determined among the 0/1, 2–4, and 5+ groups.

In all three models, estimated survival functions were generated *via* the Kaplan-Meier method and compared with the logrank test, univariate Cox analysis was used to assess the variables that affect survival significantly. Subsequently, these variables were further validated through multivariate Cox analysis for detecting independent factors. The three models were evaluated using C-index. All statistical analyses were performed using R program version 3.4.3. Statistical significance was set at p < 0.05 (two-sided).

## Results

### Baseline data

A total of 1,689 patients were included with a mean age of 52 ± 17 years, in which 665 (39.4%) were males and 1,024 (60.0%) were females. Caucasian patients accounted for 76.1% of the total population. During the initial treatment, 59.0% of the patients were married. Low-, moderate-, and high-grade disease occurred in 427 (25.3%), 611 (36.2%), and 446 (26.4%) patients, respectively. The tumor stages were distributed as T1/2 in 604 (35.8%) patients and T3/4 in 955 (56.5%) patients. ENE was present in 49 (2.9%) patients. Distant metastasis was present in 90 (5.3%) patients during diagnosis. Total parotidectomy was performed in 1,157 (68.5%) patients. A total of 1,294 (76.6%) and 107 (6.3%) patients received radiotherapy and chemotherapy, respectively.

LN metastasis occurred in 534 (31.6%) patients, in which 282 patients had one positive LN, 108 patients had two positive LNs, 62 patients had three positive LNs, 24 patients had four positive LNs, 16 patients had five positive LNs, and 42 patients had six or more positive LNs.

### Univariate Cox analysis

**Table 1** presents the potential predictors of DSS. Compared to low-grade disease, moderate- and high-grade disease was associated with increased one- and two-fold risk of cancer-caused death, respectively. T3/4 tumors had an HR of 1.95 (95% CI: 1.01–3.26), which was statistically higher than that in T1/2 tumors (p < 0.001). Distant metastasis predicted an HR of 1.77 (95% CI: 1.11–2.46) of cancer-caused death. A statistical relationship between age, sex, race, marital, ENE, operation type, radiotherapy, chemotherapy, and TTS and DSS was not noted (all p > 0.05).

**Table 1:** Univariate cox analysis of the impact of clinicopathologic variables on disease specific survival.

Variable	p	HR [95%CI]
Age		
<60 (n=1072)		
60-69 (n=335)	0.260	1.15 [0.90-1.45]
70+ (n=282)	0.154	0.81 [0.61-1.08]
Sex		
Male (n=665)		
Female (n=1024)	0.867	0.98 [0.81-1.20]
Race		
White (n=1286)		
Black (n=183)	0.386	1.14 [0.84-1.55]
Others (n=220)	0.618	0.93 [0.69-1.25]
Marital		
Married (n=996)		
Single (n=328)	0.994	1.00 [0.78-1.29]
Others (365)	0.793	0.97 [0.76-1.23]
Grade		
Low (n=427)		
Moderate (n=611)	<0.001	1.89 [1.03-2.99]
High (n=446)	<0.001	2.79 [1.76-4.87]
Tumor stage		
T1+T2 (n=604)		
T3+T4 (n=955)	<0.001	1.95 [1.01-3.26]
Extranodal extension (n=49)	0.177	4.83 [0.77-19.55]
Distant metastasis (n=90)	<0.001	1.77 [1.11-2.46]
Operation type	0.491	1.08 [0.87-1.33]
Non-total (n=532)		
Total (n=1157)		
Radiotherapy (n=1294)	0.859	0.98 [0.78-1.24]
Chemotherapy (n=107)	0.076	1.40 [0.97-2.03]
TTS*(months)		
<3 (n=1422)		
3+ (n=267)	0.424	0.77 [0.41-1.47]

*TTS, time to surgery.

**Table 2** presents the potential predictors of OS. Compared to the younger ones, patients aged 70+ had an additional nearly three-fold risk of overall death. Both moderate- and high-grade disease statistically meant more risk of overall death than low-grade disease (both p < 0.001). T3/4 tumors had an HR of 2.92 (95% CI: 1.02–5.08), which was statistically higher than that in T1/2 tumors (p < 0.001). Distant metastasis predicted an HR of 2.89 (95% CI: 1.54–5.44) of overall death. A statistical relationship between sex, race, marital, ENE, operation type, radiotherapy, chemotherapy, and TTS and OS was not noted (all p > 0.05).

**Table 2:** Univariate cox analysis of the impact of clinicopathologic variables on overall survival.

Variable	p	HR [95%CI]
Age		
<60 (n=1072)		
60-69 (n=335)	0.138	1.16 [0.95-1.42]
70+ (n=282)	<0.001	3.85 [1.58-8.09]
Sex		
Male (n=665)		
Female (n=1024)	0.663	0.96 [0.82-1.14]
Race		
White (n=1286)		
Black (n=183)	0.478	1.10 [0.86-1.42]
Others (n=220)	0.569	0.93 [0.72-1.19]
Marital		
Married (n=996)		
Single (n=328)	0.895	0.99 [0.80-1.22]
Others (365)	0.979	1.00 [0.82-1.22]
Grade		
Low (n=427)		
Moderate (n=611)	<0.001	1.27 [1.09-2.02]
High (n=446)	<0.001	2.18 [1.61-3.70]
Tumor stage		
T1+T2 (n=604)		
T3+T4 (n=955)	<0.001	2.92 [1.02-5.08]
Extranodal extension (n=49)	0.084	3.42 [0.85-13.81]
Distant metastasis (n=90)	<0.001	2.89 [1.54-5.44]
Operation type		
Non-total (n=532)		
Total (n=1157)	0.271	1.10 [0.93-1.32]
Radiotherapy (n=1294)	0.803	1.03 [0.84-1.25]
Chemotherapy (n=107)	0.081	1.33 [0.97-1.82]
TTS*(months)		
<3 (n=1422)		
3+ (n=267)	0.308	0.92 [0.77-1.08]

*TTS, time to surgery.

### Impact of the number of positive LNs

**Figure 2:**
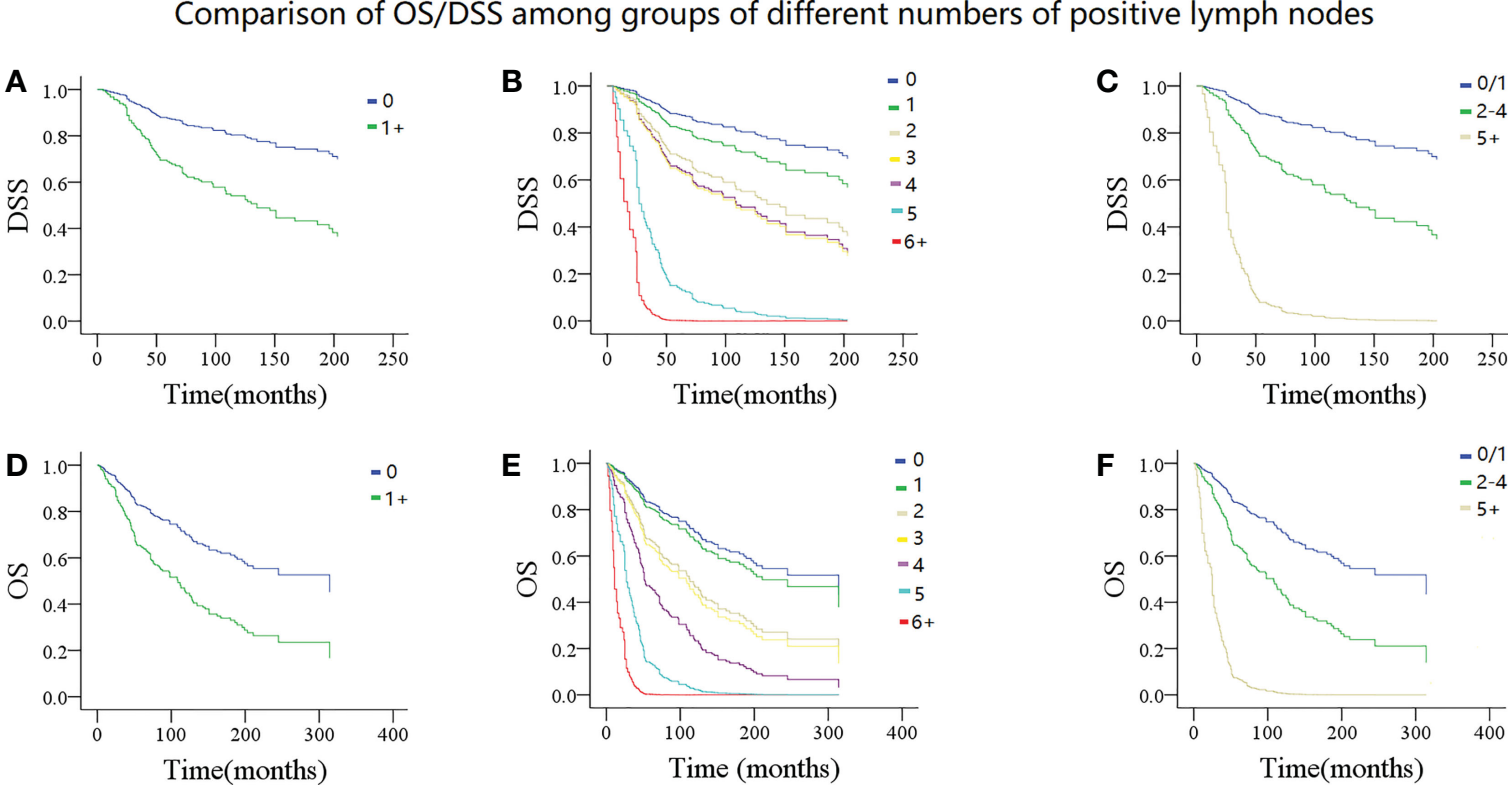
Comparison of disease specific survival (DSS) and overall survival (OS) in patients with different numbers of positive lymph nodes. DSS: **(A)** 0 vs 1+; **(B)** 0 vs 1 vs 2 vs 3 vs 4 vs 5 vs 6+; **(C)** 0/1 vs 2-4 vs 5+; OS: **(D)** 0 vs 1+; **(E** 0 vs 1 vs 2 vs 3 vs 4 vs 5 vs 6+; **(F)** 0/1 vs 2-4 vs 5+.

In model 1, the presence of LN metastasis statistically decreased DSS and OS in the univariate and multivariate Cox analyses and was associated with an additional nearly two- and 1.5-fold risk for cancer-caused and overall death, respectively (**Tables 3**, **4**). The 10-year DSS rates for patients with none and 1+ positive LN were 76% (95% CI: 72%-80%) and 52% (95% CI: 46%-58%), respectively, the difference was significant (p<0.001). The 10-year OS rates for patients with none and 1+ positive LN were 65% (95% CI: 61%-69%) and 43% (95% CI: 37%-49%), respectively, the difference was significant (p<0.001) (**Figures 2A, D**). The C-index for DSS and OS was 0.63 (95% CI: 0.57–0.69) and 0.64 (95% CI: 0.58–0.70), respectively.

**Table 3:** Univariate and multivariate Cox analysis of the impact of number of positive lymph nodes on disease specific survival.

Classification	Univariate		Multivariate	
	p	HR [95%CI]	p	HR [95%CI]
Model 1				
0 (n=1155)			Ref	
1+ (n=534)	<0.001	2.60 [2.14-3.15]	<0.001	2.82 [1.82-4.37]
Model 2				
0 (n=1155)			Ref	
1 (n=282)	<0.001	2.13 [1.67-2.71]	0.379	1.53 [0.60-3.91]
2 (n=108)	<0.001	2.50 [1.83-3.43]	<0.001	2.75 [1.61-4.69]
3 (n=62)	<0.001	2.05 [1.27-3.33]	0.047	3.46 [1.08-14.33]
4 (n=24)	<0.001	3.00 [1.67-5.37]	0.045	3.34 [1.03-10.90]
5 (n=16)	<0.001	14.01 [8.07-24.31]	<0.001	15.19 [4.43-52.07]
6+ (n=42)	<0.001	5.90 [3.97-8.78]	<0.001	48.69 [10.33-229.45]
Model 3				
0/1 (n=1437)			Ref	
2-4 (n=194)	<0.001	1.92 [1.55-2.38]	<0.001	2.81 [1.73-4.56]
5+ (n=58)	<0.001	6.43 [4.62-8.94]	<0.001	20.15 [7.50-54.18]

**Table 4:** Univariate and multivariate Cox analysis of the impact of number of positive lymph nodes on overall survival.

Classification	Univariate		Multivariate	
	p	HR [95%CI]	p	HR [95%CI]
Model 1				
0 (n=1155)				
1+ (n=534)	<0.001	2.06 [1.75-2.42]	<0.001	2.26 [1.56-3.27]
Model 2				
0 (n=1155)				
1 (n=282)	<0.001	1.81 [1.48-2.22]	0.739	1.16 [0.50-2.69]
2 (n=108)	<0.001	1.95 [1.48-2.57]	0.029	2.37 [1.28-6.72]
3 (n=62)	0.002	1.89 [1.27-2.82]	0.001	2.16 [1.37-3.40]
4 (n=24)	0.026	1.93 [1.08-3.44]	0.001	4.12 [1.77-9.59]
5 (n=16)	<0.001	9.71 [5.64-16.72]	<0.001	10.67 [3.21-35.49]
6+ (n=42)	<0.001	3.95 [2.72-5.76]	<0.001	32.15 [7.23-143.03]
Model 3				
0/1 (n=1437)				
2-4 (n=194)	<0.001	1.69 [1.41-2.02]	<0.001	2.36 [1.58-3.54]
5+ (n=58)	<0.001	4.48 [3.27-6.14]	<0.001	14.20 [5.45-36.97]

In model 2, the univariate Cox analysis reported the statistical association between DSS/OS and the number of positive LNs. In the multivariate Cox analysis, compared with no LN metastasis, one positive LN did not provide additional compromise to DSS, while two or more positive LNs were related to worse DSS. The 10-year DSS rates of the 2, 3, and 4+ LN groups were 51% (95% CI: 41–61%), 54% (95% CI: 36–72%), and 50% (95% CI: 30–70%), respectively. Their HR was comparable, and their 95% CI greatly overlapped. In the 5 and 6+ positive LNs groups, the median DSS time was 37.0 (95% CI: 31.7–42.3) and 25.0 (95% CI: 9.3–40.6) months, respectively. Their 95% CI also apparently overlapped. As for the OS, a similar trend was observed (**Tables 3**, **4**; **Figures 2B, E**). The C-index for DSS and OS was 0.77 (95% CI: 0.73–0.81) and 0.76 (95% CI: 0.75–0.78), respectively.

In model 3, the univariate Cox analysis described a statistical association between DSS/OS and the number of positive LNs. The 10-year DSS rates in the 0/1, 2–4, 5+ positive LN groups were 74% (95% CI: 70–78%), 60% (95% CI: 54–66%), and 50% (95% CI: 48–52%), respectively, the difference was significant (p<0.001). The 10-year OS rates in the 0/1, 2–4, 5+ positive LN groups were 63% (95% CI: 59–67%), 50% (95% CI: 44–56%), and 9% (95% CI: 1–17%), respectively, the difference was significant (p<0.001) (**Figures 2C, F**). In the multivariate Cox analysis, compared with the 0/1 positive LN group, the 2–4 positive LN group had an HR of 2.81 (95% CI: 1.73–4.56) for DSS and 2.36 (95% CI: 1.58–3.54) for OS, respectively. The 5+ LN group had an HR of 20.15 (95% CI: 7.50–54.18) for DSS and 14.20 (95% CI: 5.45–36.97) for OS, respectively (**Tables 3**, **4**). The C-index for DSS and OS was 0.83 (95% CI: 0.81–0.85) and 0.82 (95% CI: 0.80–0.84), respectively.

## Discussion

The main finding was that LN metastasis was an important prognostic factor for both DSS and OS; however, the negative impact was only observed when at least two positive LNs were present. The three prognostic categories based on the number of positive LNs (0/1 vs 2–4 vs 5+) could predict oncologic outcomes in parotid ACC without overlap and ultimately help triage high-risk patients who may benefit from more aggressive adjuvant therapies.

Model 1 confirmed that LN metastasis is an independent predictor of DSS and OS. However, it referred to the metastasis of intraparotid or neck LN, both of which significantly decrease survival. Han et al. (10) analyzed the association between cervical LN involvement and OS in 54 patients and found that node status was the only independent prognostic factor. Moreover, neck LN metastasis was related to nearly an increased five-fold risk of overall death. Feng et al. (11) discussed the significance of intraparotid LN metastasis in 337 patients and reported that the 10-year local control rate was 94% for patients without intraparotid LN metastasis, 56% for patients with metastasis in no more than two intraparotid LNs, and 22% for patients with metastasis in more than two intraparotid LNs. Moreover, the differences were statistically significant independently. However, model 1 could not explain the effect of LN metastasis on survival as stratified through numbers accurately.

Model 2 provided a more interesting finding. Firstly, this was the first to report that the presence of only one positive LN did not pose any additional compromise in survival compared with the absence of LN metastasis. In many solid cancers, prognosis would be decreased by up to half although there was only one positive LN (12). The obvious difference might be accounted by the unique features of ACC, and that the common cause of death was distant metastasis rather than regional LN metastasis (13). Moreover, LN metastasis was relatively infrequent in ACC (14). This finding offered new insights into the clinical management of parotid ACC. Secondly, the negative impact of LN metastasis on DSS or OS began to appear when there was at least two positive LNs, and the effect did not increase significantly although four positive LNs were detected. A few studies aimed to clarify how different numbers of positive LNs affect survival in ACC. Liu et al. (15) analyzed the outcome of 47 patients with pN+ ACC in the head and neck. They found that in cases with one, two to three, and four positive LNs, the 5-year OS rates were 86.6%, 66.3%, and 60.0%, respectively, and the difference was not significant. This difference from the current study could be contributed by their small sample size and different cutoff values. However, another study reported that a positive LN ratio greater than 0.2 was associated with poorer metastasis-free survival, and a ratio > 0.07 predicted worse DSS (16). However, the ratio was greatly decided by the dissection of the LN, which may be influenced by several uncontrollable factors. Moreover, the method of LN examination varied among different medical centers, and the number, as a variable, was likely to be more stable than the ratio. Thirdly, DSS and OS were greatly inhibited if five or six or more positive LNs were present, and the 95% CI of the survival rates and HR of the two groups apparently overlapped. A study which focused on salivary duct carcinoma (17) reported that the presence of nine or more positive LNs had increased the nearly 11-fold risk of overall death compared to eight or less positive LNs. They also reported in another study that patients with five or more positive LNs were significantly at higher chance of developing cancer-caused death in major salivary gland carcinoma (18).

Model 3 offered the best predictive value for DSS and OS. Its cutoff was determined based on models 1 and 2 with high rationality and reliability, and the three groups had distinct, non-overlapping prognosis and HR. Only a few studies aimed to propose a new LN staging system that will be beneficial for clinical practice. In a study consisting of 307 patients treated for salivary gland carcinoma (6), ENE did not exhibit any negative impact on DSS, OS, locoregional recurrence, or distant metastasis. Moreover, the neck stage based on the 8^th^ AJCC classification could not present a satisfactory OS stratification. However, the new LN system, which was developed based on the number of positive LNs (0 vs 1–3 vs 4+) and/or their maximum diameter (< 20 mm vs 20+ mm) showed better accuracy in OS prediction. In another similar study (7), ENE was also not related to worse OS, and a four-category LN staging system according to the number of positive LNs (0 vs 1–2 vs 3–21 vs 22+) was superior to the current N classification at OS stratification. Boon et al. (19) evaluated the results of 177 patients with salivary duct carcinoma and reported that the absolute number of metastatic LNs (0 vs 1–2 vs 3–15 vs 16+), rather than the traditional cervical stage, was the only significant prognostic factor for OS as shown by the results of a multivariate analysis.

Therefore, four key points could be deduced: (1) ENE may demonstrate little influence on survival; (2) intraparotid LN should be taken into consideration in nodal staging; (3) an LN staging based on the number of positive LNs could be a good surrogate for the current system; and (4) the optimal cutoff for the number of positive LNs varied with the histologic type. Our three prognostic categories provided an accurate discrimination for low-, moderate-, and high-risk patients and could be used as a central predictor of mortality in parotid ACC.

Nevertheless, this study still has some limitations. First, this was a retrospective study; hence, there may be inherent bias. Second, data on lymphovascular invasion and margin status were not available. Third, we only enrolled patients with parotid ACC, it remained unknown whether current finding was suitable for parotid cancers of other histologic types.

In summary, LN metastasis significantly impacts survival in parotid ACC; however, the effect is not apparent until at least two positive LNs are present. Our three prognostic categories based on the number of positive LNs (0/1 vs 2–4 vs 5+) could be used to screen patients with different risks and plan for more aggressive treatment for high-risk patients.

## Data availability statement

The datasets presented in this study can be found in online repositories. The names of the repository/repositories and accession number(s) can be found in the article/supplementary material.

## Ethics statement

Ethics approval was not required owing to public access of the data.

## Author contributions

The author confirms being the sole contributor of this work and has approved it for publication.
